# CFTR, Cell Junctions and the Cytoskeleton

**DOI:** 10.3390/ijms23052688

**Published:** 2022-02-28

**Authors:** Ines Pankonien, Margarida C. Quaresma, Cláudia S. Rodrigues, Margarida D. Amaral

**Affiliations:** BioISI—Biosystems and Integrative Sciences Institute, Faculty of Sciences, University of Lisboa, 1749-016 Lisboa, Portugal; ipankonien@fc.ul.pt (I.P.); mcquaresma@fc.ul.pt (M.C.Q.); cadrodrigues@fc.ul.pt (C.S.R.)

**Keywords:** cystic fibrosis, cell polarization, epithelial differentiation, epithelial regeneration, epithelial-mesenchymal transition, extracellular matrix

## Abstract

The multi-organ disease cystic fibrosis (CF) is caused by mutations in the gene encoding the CF transmembrane conductance regulator (CFTR) protein, a cAMP regulated chloride (Cl^−^) and bicarbonate (HCO_3_^−^) ion channel expressed at the apical plasma membrane (PM) of epithelial cells. Reduced CFTR protein results in decreased Cl^−^ secretion and excessive sodium reabsorption in epithelial cells, which consequently leads to epithelial dehydration and the accumulation of thick mucus within the affected organs, such as the lungs, pancreas, gastrointestinal (GI) tract, reproductive system and sweat glands. However, CFTR has been implicated in other functions besides transporting ions across epithelia. The rising number of references concerning its association to actin cytoskeleton organization, epithelial cell junctions and extracellular matrix (ECM) proteins suggests a role in the formation and maintenance of epithelial apical basolateral polarity. This review will focus on recent literature (the last 10 years) substantiating the role of CFTR in cell junction formation and actin cytoskeleton organization with its connection to the ECM.

## 1. Introduction

A proper physical barrier function is key to a structured epithelium and this property is mediated by different cell–cell junctions that extracellularly link the epithelial cells together while providing intracellular connections with different elements of the cytoskeleton in a functional continuum [1]. Epithelial cells contact each other through subapical tight junctions (TJs) and adherens junctions (AJs), while gap junctions (GJs) and desmosomes provide basolateral cell–cell contacts [2]. These cell junctions tightly connect epithelial cells together, while allowing them to communicate with each other [3]. TJs and AJs are particularly important due to their direct connection to the cytoskeleton through actin and microfilaments, as well as to polarity protein complexes, establishing and maintaining an apical basolateral polarity critical for proper epithelial cell function [1,4,5].

Alterations in epithelial organization and function can occur in several pathological conditions, including chronic inflammatory diseases [6,7]. Such is the case with Cystic Fibrosis (CF), a monogenic recessive disease caused by mutations in the CFTR gene, which encodes the CF Transmembrane Conductance Regulator (CFTR) protein. CFTR is a cAMP-activated, ATP-gated Cl^−^ and HCO_3_^−^ channel expressed at the apical plasma membrane (PM) of polarized epithelial cells, where it regulates ion flux in order to maintain the correct water and salt balance at the surface epithelia [8]. The most common mutation in CF individuals, F508del, results in impaired CFTR protein folding, PM trafficking, function, and PM stability. Defective CFTR-mediated ion transport leads to severe dehydration and mucus accumulation within the affected organs, such as the pancreas, gastrointestinal (GI) tract, reproductive system and sweat glands, although it mainly affects the airways. The thick and sticky mucus in the lungs favours recurrent respiratory infections and progressive loss of lung function, ultimately leading to respiratory failure [9]. However, many studies have reported, as ‘secondary’ effects of CFTR dysfunction, that CF epithelia display a disorganized actin cytoskeleton [10,11,12] and high paracellular permeability in parallel with low transepithelial electrical resistance (TEER). All these features are consistent with disrupted tight junction (TJ) structure [13,14,15,16], thus suggesting that CFTR has important roles in the maintenance of the proper structure and function of the epithelia. In fact, there are several important lines of evidence that connect CFTR to the cytoskeleton, cellular junctions and even the extracellular matrix (ECM).

In this review we aim to summarize the complex body of evidence published in the last 10 years regarding the interactions between CFTR, actin cytoskeleton, cell junctions and ECM. Herein, we intend to promote our rethinking about the way CF is perceived. We aim to establish that CF is not ‘merely’ a channelopathy/ion channel disease, but also a disorder of epithelial differentiation, particularly evident at the level of the structure and function of the cytoskeleton and cell junctions, as previously proposed [17].

## 2. CFTR and the Actin Cytoskeleton

Several studies have described the interplay between CFTR and the actin cytoskeleton, ranging from the importance of functional CFTR for the integrity of the cytoskeleton to the impact of the cytoskeleton on CFTR surface expression and function in epithelial cells. In this section we dissect the most important and recent findings of this intriguing relationship.

Intrinsic to their polarized organization, epithelial cells display microdomain compartmentalization of multiprotein complexes comprising membrane receptors, intracellular signalling messengers, effectors, and scaffolding molecules. This allows for local regulation of apical or basolateral transporters and facilitates signal transduction with increased specificity and efficiency [18,19,20]. As an apically expressed ion channel in polarized epithelia, CFTR surface expression and function depends on a high level of cytoskeletal fibrillar (F)-actin organization [9]. Both N- and C-termini of CFTR are intracellularly located and thus facilitate its binding to several cytoplasmic partners including other ion channels and transporters, receptors, kinases, phosphatases, signalling molecules and cytoskeletal elements [21,22].

### 2.1. NHERF1-Ezrin-Actin Complex

The most studied cytoskeletal protein that associates with CFTR is the Na^+^/H^+^ exchanger regulatory factor isoform 1 (NHERF1, also known as SLC9A3R1, EBP50). The C-terminus of CFTR harbours a PDZ-containing domain which was found to interact with the PDZ domain of NHERF1 thereby tethering CFTR to the apical PM [23]. In addition, the overexpression of NHERF1 has been shown to partially rescue F508del-CFTR to the apical surface of CF epithelial cells. NHERF1 overexpression also increased fibrillar (F)-actin organization which in turn led to the binding of F508del-CFTR to the cytoskeleton, thereby stabilizing it to the PM. This further delayed its internalization fostering fine-tuning of CFTR channel function and physical association with different signal transduction players [11,14].

**Figure 1:**
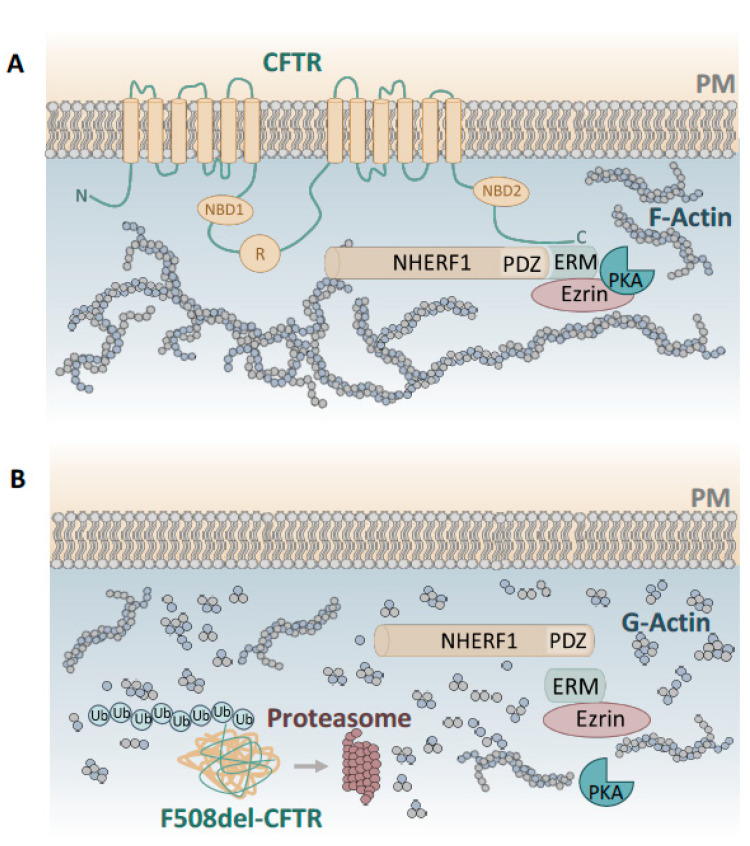
CFTR and its connections to the actin cytoskeleton. (**A**) In normal epithelial cells CFTR is expressed at the apical plasma membrane (PM) and promotes the polymerization of actin monomers needed to establish a well-organized actin cytoskeleton. (**B**) In CF cells, the actin cytoskeleton is less organized due to dysfunctional CFTR, which is not transported to the apical PM, but targeted for degradation. Adapted from [23].

Stimulation of endogenous small GTPase Rac1 signalling (via hepatocyte growth factor, HGF) was shown to retain F508del-CFTR at the PM of primary human bronchial epithelial (HBE) cells by promoting its NHERF1-mediated anchoring to the actin cytoskeleton [24,25,26]. In fact, NHERF1 regulates CFTR stability and function at the PM by forming a regulatory protein macro-complex which also includes the structural protein ezrin, a member of the Ezrin, Radixin and Moesin (ERM) protein family which is linked to actin. NHERF1 binds to CFTR through its PDZ domain and ezrin via its ERM binding domain and together they form the CFTR-NHERF1-Ezrin-actin protein complex which stabilizes CFTR at the apical PM (Figure 1). Ezrin as a PKA-anchoring protein also provides a link to PKA in the proximity of CFTR, thereby regulating cAMP-dependent Cl^−^ efflux, reviewed in [17].

Importantly, it has been found that, in non-CF airway epithelial cells, NHERF1 and ezrin are mostly located close to the apical region, while in CF cells, they rather localize to the cytoplasm, which is suggestive of the fact that somehow, CFTR must promote the polymerization of actin monomers needed to establish a well-organized actin cytoskeleton (Figure 1). This is supported by several studies that observed a more disorganized actin cytoskeleton, with disrupted or absent actin stress fibres in CF cells when compared to non-CF cells [10,11,12,27,28]. Moreover, CFTR silencing has been shown to promote the loss of cortical actin filament and disruption of cAMP compartmentalization at the apical PM, further supporting a role for CFTR in cytoskeletal organization during epithelial cell polarization [17,18].

The use of F508del-CFTR correctors Trimethylangelicin (TMA) and VX-809 in CF cells partially restored the actin cytoskeleton organization [29], indicating a role of CFTR (not necessarily functional) at the PM in the correct assembly of the cytoskeleton, which is essential for cell shape and function. However, cytoskeletal disruption has long been shown to greatly affect the cAMP-mediated activation of CFTR, suggesting their interplay [22,23]. A striking open question remaining to be addressed is whether the new CFTR correctors VX-445 and VX-661 are also able to rearrange actin while rescuing the role of mutant CFTR as an ion channel.

Importantly, wt-CFTR knockdown alone is sufficient to promote loss of cortical actin filaments and disrupt cAMP compartmentalization, suggesting that expression of CFTR at the apical PM is essential not only for the formation of the CFTR-NHERF1-ezrin-actin complex but also for the integrity of the apical compartment itself, once again hinting at CFTR as a key player in epithelial cellular organization [22]. Further evidence was found by Watson et al. [27]; by removing the PDZ binding motif of CFTR, these authors found that the rearrangement of actin was prevented. Moreover, a discrete wt-CFTR localization at the PM was detected by super resolution microscopy, distinct from intracellular CFTR and closely correlated (co-localized) with the actin cytoskeleton. However, this was not the case for the truncated form of CFTR where the PDZ domain was removed, thus further highlighting the association of CFTR with actin through its C-terminus [27].

Another study by Trouvé and colleagues on G551D-CFTR, a class III mutation with normal cell surface expression but with impaired channel activity, reported that it binds more to actin in comparison to wt-CFTR and that this is needed for its stabilization at the PM. However, this did not favour its function, as with the disruption of actin, the activation of the weak G551D-CFTR Cl^−^ currents in patch-clamp experiments was also lost [30]. This proves that actin is necessary for CFTR functioning, but also indicates that local Cl^−^ and/or HCO_3_^−^ transport is required for a correct actin cytoskeleton organization. A very recent study investigated the role of intracellular Cl^−^ concentration in bronchial epithelial cells (16HBE) and found that local Cl^−^ accumulation promoted F-actin reorganization. Results showed that peripheral F-actin fibres were disassembled while the amounts of threadlike stress fibres were markedly increased, suggesting that Cl^−^ affects the cellular properties of airway epithelial cells [31]. However, it was not investigated how actin would be affected under high Cl^−^ concentrations in cells expressing CFTR mutants.

### 2.2. Other Cytoskeleton Regulators

Besides the well-studied NHERF1-ezrin complex anchoring CFTR to the actin cytoskeleton, other cytoskeleton-related proteins have been identified which possibly indirectly regulate CFTR, and these are reviewed in this section. Recently, Family with sequence similarity 13 member A (FAM13A) has been identified as a modifier gene of the CF lung phenotype [32]. FAM13A contains a GTPase-activating (GAP) domain and GTPases are known to play a role in the actin cytoskeleton and remodelling, as they regulate the assembly of F-actin stress fibres [33]. This study further investigated the role of FAM13A in CF and found that its expression is decreased in primary HBE cells from CF individuals compared to controls. An additional silencing of FAM13A in CF primary HBEs was associated with changes in the F-actin cytoskeleton through RhoA activity [32]. These findings suggest that FAM13A downregulation contributes to the observed decrease in organization of the actin cytoskeleton in CF cells. It would be important to show whether overexpression of FAM13A aids the assembly of actin fibres and, thereby, possibly rescues mutant CFTR.

EPAC1 (also known as RAPGEF3) which is known to be involved in actin cytoskeleton rearrangements and cell polarization has also been identified as a CFTR-interacting protein [34,35]. EPAC1 has been shown to be activated by high levels of cAMP which promotes its interaction with NHERF1, thus stabilizing CFTR at the PM [34] and most likely also contributing to the polarized organization of epithelial cells. A follow-up study identified two new cytoskeleton regulators, CAPZA2 and INF2, which have been found to associate with CFTR under EPAC1 activation. CAPZA2, a capping protein, binds the barbed end of actin filaments while also forming a stabilized complex with CFTR and EPAC1 close to the PM. INF2 also binds to the actin filaments regulating actin cytoskeleton dynamics, thereby negatively influencing CFTR anchoring to the PM. The authors further suggest that both proteins together balance the regulation of actin cytoskeleton dynamics and CFTR PM stability [36].

Taken together, all these data emphasize a strong connection between CFTR and the actin cytoskeleton and/or cytoskeletal regulators, and that the establishment of a well-organized actin cytoskeleton is lost in CF when CFTR is absent or dysfunctional. It is important to further study the mechanisms by which CFTR and its interactors regulate the actin cytoskeleton (and vice versa) in order to identify new possible drug targets for CF.

## 3. CFTR and Cell Junctions

### 3.1. Epithelial Tightness: Transepithelial Electrical Resistance (TEER) and Tight Junctions (TJs)

Given the fact that actin filaments are directly connected to intracellular cell junctions, playing an important role in the assembly and maintenance of these structures, they also provide a link between CFTR and cell junctions [37]. Therefore, it is perhaps not surprising that CFTR plays a role in cell junction formation and vice versa, i.e., CFTR localization is regulated by junctional proteins. In fact, TJs are tightly connected to the apical actin network and restrict the paracellular diffusion of small solutes and fluid across the intercellular space of the epithelium. They determine the boundary between the apical and basolateral membranes and define epithelial tightness, thereby strongly regulating the transepithelial resistance of polarized cells. Indeed, it has been found that CFTR at the PM is essential for the correct structural organization and function of TJs and consequently for correct epithelial barrier function and tightness of epithelial cells, namely those lining the airways. As shown in several studies, cells expressing F508del-CFTR exhibit decreased epithelial tightness compared to wt-CFTR expressing cells, as demonstrated by the lower transepithelial electrical resistance (TEER) of F508del-CFTR versus wt-CFTR expressing cells [14,15,38,39,40]. Furthermore, permanent or temporary downregulation of wt-CFTR led to a reduction in TEER values, thus supporting the idea that CFTR promotes epithelial tightness/polarization [16,28].

Interestingly, we recently found that knocking-out (KO) KLF4, a transcription factor known to regulate epithelial differentiation among other processes [41,42], in polarized wt-CFTR-expressing bronchial epithelial (CFBE) cells leads to a significant decrease in TEER. The opposite was seen in F508del-CFTR-expressing cells upon KLF4-KO which led to an increase in TEER levels compared to control cells, albeit not reaching wt-CFTR TEER levels [43]. This suggests that KLF4 is needed for epithelial integrity/polarization of normal cells expressing wt-CFTR. However, in the case of CF cells, where epithelial tightness is already weakened (i.e., the cells are more mesenchymal), the loss of KLF4 counteracts the reduced resistance.

Intriguingly, a recent study characterized a novel, more physiological, co-culture of CFBE wt-CFTR/F508del-CFTR cells with human vascular endothelial cells. The authors observed tighter barrier properties, as demonstrated by the increased TEER and decreased permeability in F508del-CFTR CFBE cells co-cultured with endothelial cells compared to mono-cultures [44]. As shown by several studies, the lower TEER found in cells expressing F508del-CFTR is accompanied by an increase in paracellular permeability and a disruption of the TJ structure with disorganization of TJ proteins [13,14,15,38,39] which normally localize to the apical region in epithelial cells. De Lisle analysed the TJ composition in the intestine of a CFTR knockout mouse [13]. The mRNA expression levels of several claudins (namely, 1, 2, 3, 5, 7, and 8), which are integral membrane proteins of TJs normally localized at the apico-lateral surface, were altered and the localization of these proteins at the apical level was either reduced or lost, with their mislocalization to the basal side of the villi enterocytes. A very similar pattern was found for occludin, another TJ protein member, being absent at the lateral side of CF cells with an accumulation in the cytoplasm on the basal side. A more recent transcriptomic analysis of the distal small intestine of CFTR null mice also revealed differential regulation of genes involved in intestinal barrier function [45]. Gene set enrichment analysis showed differential expression of genes representing the apical junction complex, including claudins-2,-3, and -8 which were found to be reduced in CFTR null mice. Occludin was also found to be reduced, but only marginally. However, the authors emphasize that they found WNK4 protein kinase to be consistently reduced among their analysed samples which could be of relevance since WNK4 phosphorylates specific claudins at the TJs and their activation enhances paracellular Cl- permeability [45,46].

Additionally, in cholangiocytes from CFTR null mice, another TJ protein, zonula occludens 1 (ZO-1), appeared diffusely distributed in the cytoplasm as observed by the significant increase in the cytoplasmic/junctional fluorescence ratio in CF compared to the control [28]. These data indicate an abnormal structure of TJs in mouse intestine due to the absence of CFTR. Furthermore, these observations are supported by studies in human airway epithelial cells comparing CFBE (CF) with 16HBE (non-CF) cells. While 16HBE cells showed the distinctive chicken-wire patterns of TJ proteins ZO-1, occludin, claudin-1, and junction-associated adhesion molecule 1 (JAM-1) with their typical apical localization, in CFBE cells this organized protein expression pattern was found to be disturbed. ZO-1 and occludin shifted to the cytoplasm and the nucleus, claudin-1 was poorly expressed at the PM and JAM1 was barely detectable in CFBE cells. Interestingly, the ZO-1 expression pattern at the TJ level in CFBE cells could be partially rescued by NHERF1 overexpression, but also by wt-CFTR overexpression, in parallel with decreased paracellular permeability. These data indicate that the protein complex CFTR-NHERF1-ezrin-actin plays an important role in TJ structure and function, suggesting a possible physical interaction with TJ components [14].

We recently investigated epithelial–mesenchymal transition (EMT), which had been suggested to occur in CF; it is a process which involves the disruption of cell junctions, cell adhesion, cell polarity, remodelling of the cytoskeleton, and changes in cell–matrix adhesion. We used polarized CFBE cell lines overexpressing wt- or F508del-CFTR (which have the advantage of being isogenic) so as to directly link defective CFTR to any observed differences. Considering TJs, in Western blot analysis, ZO-1 protein expression was found to be increased while claudin-1 showed decreased expression in F508del-CFTR cells vs. wt-CFTR cells. Although the increased ZO-1 expression seemed to contradict other studies, when observing its subcellular localization by immunofluorescence in CF cells, we found a highly disorganized pattern compared to the nicely defined TJ staining in non-CF cells, which was in agreement with previous studies. A likely explanation for the ZO-1 upregulation in CF cells is that, possibly through a feedback mechanism, ZO-1 expression is stimulated to compensate for the absence of functional ZO-1 [40]. That CFTR indeed directly interacts with TJ protein ZO-1 through its PDZ-binding domain was shown in a study using the epididymis as a model system. This interaction modulates proliferation and differentiation genes under the control of the ZO-1/ZONAB pathway suggesting that CFTR regulates TJ formation and thereby also promotes cell differentiation [16]. Interestingly, CFTR also affects TJ protein ZO-1 in human keratinocytes. Indeed, it has been shown that downregulation of CFTR disrupts the formation of cell junctions and vice versa [47].

In conclusion, the data summarized here highlight the relation between epithelial tightness, represented by high TEER, and proper tight junction assembly, with CFTR, and the loss of those epithelial structures in CF cells.

### 3.2. Gap Junctions (GJ), Adherens Junctions (AJ), and Desmosomes

CFTR is also deeply associated with GJs, AJs, and desmosomes which are reviewed here. Similarly to TJs, AJs serve as subapical cell-to-cell contacts between epithelial cells. GJs and desmosomes provide lateral cell-to-cell contacts [2]. AJs are functionally highly interdependent and crucial in regulating a set of polarity protein complexes that confer upon epithelial cells their characteristic apical-basal polarity [48]. While GJs mediate cellular communication by forming intercellular channels that promote diffusion of ions and metabolites between cells (and across cell layers), desmosomes are important for the resistance of epithelial tissues to mechanical stress, and for interactions with intermediate filaments [5,39].

Molina et al. used NuLi-1 (CFTR^wt/wt^) and CuFi-5 (CFTR^ΔF508/ΔF508^) cells cultured at air-liquid interface (ALI) to study cell junction complexes and their relationship with CFTR. They found that GJ protein connexin-43 (CX43) mislocalized to perinuclear regions in CuFi-5 cells, whereas NuLi-1 cells displayed a correct GJ punctuate staining along the cell borders [39]. This is supported by our own study, where we found a disorganized staining pattern for CX43 in F508del-CFTR expressing cells vs. wt-CFTR cells [40]. Additionally, in the same study, staining of the AJ proteins E-cadherin and β-catenin appeared more diffuse in CF cells compared to control cells. On the other hand, we found an increased expression of N-cadherin, a marker for mesenchymal cells, suggesting a partial EMT in CF cells [40]. Importantly, the rescue of F508del-CFTR by the current modulator drugs VX-445/VX-661 led to a significant reduction in the expression of N-cadherin, thus restoring a more epithelial phenotype, albeit only partially. Since the effect was even increased when correctors were combined with potentiator VX-770, this implies that functional CFTR at the PM (and not just its presence) is required for proper cell junction integrity and correct architecture of polarized epithelial cells [40,49]. However, the observed effect could also be a secondary consequence by which cell junctions are restored through CFTR-mediated F-actin cytoskeleton re-arrangement. Interestingly, a recent proteomic screen in human lung epithelial cells identified β-catenin (a subunit of the cadherin protein complex involved in the regulation and coordination of cell–cell adhesion) as a potential interacting protein of wt-CFTR, but not F508del-CFTR. A lack of interaction between this AJ protein and dysfunctional CFTR further supports a role for CFTR in correct AJ assembly and/or localization [50]. Furthermore, in mouse intestine, wt-CFTR was shown to stabilize β-catenin and prevent its degradation. However, in the case of F508del-CFTR, no interaction with β-catenin was observed, leading to its degradation, and supporting the previous study. Nevertheless, it would be of interest to investigate whether or not the β-catenin-CFTR interaction persists when non-functional CFTR is present at the PM (i.e., G551D-CFTR). In contrast to these observations, Grof et al. [44] observed no difference in β-catenin staining between CFBE wt- and F508del-CFTR-expressing cells in mono-culture. However, when co-cultured with human vascular endothelial cells, β-catenin staining increased in F508del-CFTR cells vs. wt-cells, suggesting improved AJ function [44].

Two other studies show that dysfunctional CFTR affects AJ formation factor (afadin) [28,51]. Sun et al. found that wt-CFTR and afadin are co-localized at apical cell contacts and interact with each other through their respective PDZ domains, in the colon cancer cell line Caco-2. Those studies report that CFTR downregulation leads to a faster degradation of afadin and to a reduction in epithelial tightness, thus proposing that CFTR has a stabilizing function on afadin [51]. In the biliary epithelium of CFTR-KO mice, it was shown that afadin loses its junctional restriction and appears diffusely distributed in the cytoplasm. Consistent with junctional defects, epithelial permeability was significantly increased while TEER was decreased in monolayers of CFTR-KO cells contributing to the loss of the integrity of the epithelial barrier function.

**Figure 2:**
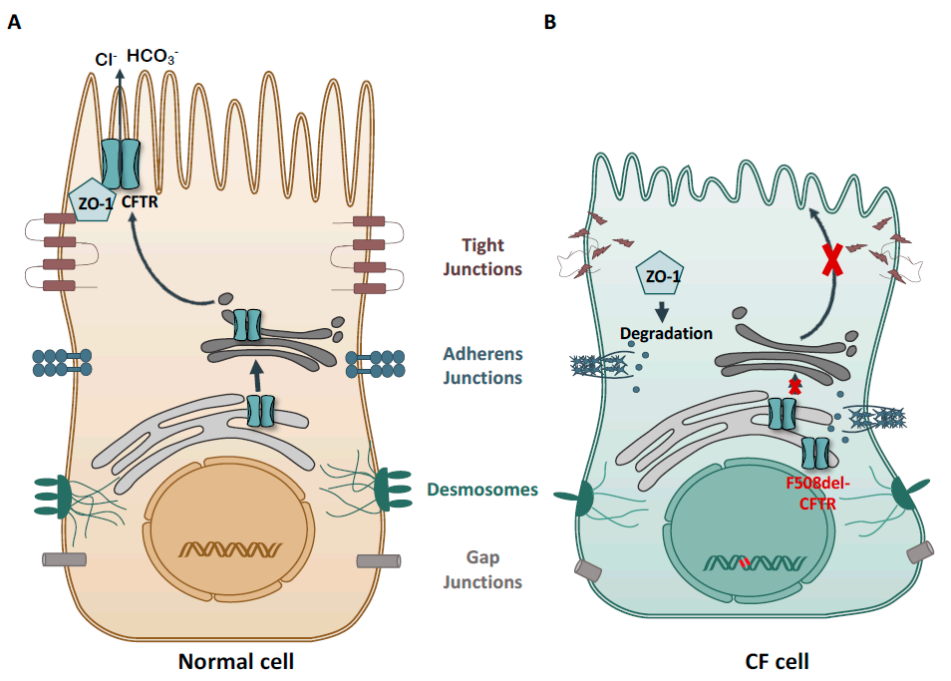
CFTR and its association with cell junctions. (**A**) In normal epithelial cells CFTR expression at the plasma membrane contributes to a correct cell junction integrity and correct architecture of polarized epithelial cells. (**B**) In CF cells, dysfunctional CFTR leads to the disruption of cell junction structures, resulting in reduced epithelial barrier function.

Indeed, we also found desmoplakin, a component of intercellular junctions called desmosomes, to be downregulated in F508del-CFTR expressing cells compared to wt-CFTR cells [40]. The overall disruption of cell junctions in CF is shown in Figure 2.

Taken together, all these data emphasize that CFTR’s association with cell junctions plays a key role in the formation of the epithelial barrier, and this is disrupted in CF cells, rendering the CF epithelium more permeable, more vulnerable to pathogens and less efficient in epithelial differentiation and regeneration after injury. As such, junctional proteins are potential new drug targets for the rescue of epithelial integrity and regeneration in CF.

## 4. CFTR, Integrins and Extracellular Matrix (ECM)

Integrins are surface receptors that mediate the link between the extracellular matrix (ECM) and the actin cytoskeleton in epithelial cells and are also essential for the establishment of cell polarity and cell adhesion [52,53]. We herein present studies which observed changes in these ECM-related proteins due to dysfunctional CFTR. Itokazu et al. [54] found that, in CFTR-silenced cells, β1-integrin is downregulated and levels of phosphorylation of focal adhesion kinase (FAK) are reduced due to a loss of gangliosides, which are sphingolipids that play a role in cell migration by their interaction with integrins. The authors suggest that the reduced β-integrin signalling leads to the defective cell migration in CF observed in a previous study [54,55]. In contrast to this, Grassmé et al. [56] found increased accumulation of β1-integrin on the luminal side of CF airway epithelial cells, although this protein is usually present at the basolateral side in normal airway cells. They propose that this accumulation is mediated by increased levels of ceramides, which form lipid platforms at the luminal pole where β1-integrin is trapped. This is supported by another study [57] showing that in CF, β1-integrin and fibronectin (another ECM protein) are luminally exposed and mediated by Vav3, a guanine exchange factor (GEF) regulating cytoskeleton remodelling. However, it is reported that the increased expression of β1-integrin and fibronectin attracts *Pseudomonas aeruginosa* adhesion to CF cells [57]. The discrepancy with the previous study [54] could be technical, since cells were grown on plates while the subsequent studies used a more physiological model with cells grown at ALI.

Furthermore, a proteomic study on the secretome in CF demonstrated that fibronectin, an ECM protein typically secreted basally in the airway epithelium, was one of the most increased and abundant proteins in CF apical secretions. The increased levels of ECM proteins in the CF secretome could be linked to the airway remodelling that is constantly occurring in CF [58]. We recently also observed that F508del-CFTR cells deposited more collagen I to the ECM compared to wt-CFTR expressing cells; this is one of the factors defining CF cells as more mesenchymal and more prone to EMT, which also drives remodelling [40].

In summary, dysfunctional CFTR also affects integrins and other ECM proteins in CF, likely through the actin cytoskeleton.

## 5. Conclusions

An emerging body of literature shows that CFTR is tightly associated with actin cytoskeleton organization and with cell junction formation in epithelial cells. This role is essential for the establishment of apical-basolateral polarity that provides a proper epithelial barrier function, which is indeed disrupted in CF cells. It is, however, unclear whether CFTR regulates the cytoskeletal and junctional organization, or the junctions and the cytoskeleton modulate CFTR or if, as is more likely, it is an interplay between both. It can, however, be assumed that disorganization of the actin structures and junctions in CF has implications on epithelial differentiation and regeneration. Therefore, further studies are needed to understand the role of CFTR within these processes to identify pathways/proteins that can serve as novel drug targets in CF. However, what emerges from a growing amount of evidence is that CF is not just an ion channel disease, but also a disorder of epithelial structure and function.

## Data Availability

Not applicable.

## Abbreviations

AJ: adherens junction; ALI: air-liquid interface; CF: cystic fibrosis; CFTR: CF transmembrane conductance regulator; ECM: extracellular matrix; EMT: epithelial–mesenchymal transition; ERM: Ezrin, Radixin and Moesin (protein family); GI: gastrointestinal (tract); GJ: gap junction; HBE: human bronchial epithelial (cells); NHERF1: Na+/H+ exchanger regulatory factor isoform 1; PM: plasma membrane; TJ: tight junction; ZO-1: zonula occludens 1 (protein).
